# What is the embodied CO_2_ cost of getting building design wrong?

**DOI:** 10.1098/rsta.2023.0238

**Published:** 2024-11-04

**Authors:** Cyrille F. Dunant

**Affiliations:** ^1^ Department of Engineering, University of Cambridge, Trumpington Street, Cambridge CB2 1PZ, UK

**Keywords:** construction, material mix, concrete, steel

## Abstract

Today, carbon and cost-efficient construction are well matched. However, in the future, as steel production is increasingly done from recycled scrap in electric arc furnaces (EAFs) and concrete mix design is improved, the current balance of CO_${,}_{2}$_ impacts and costs can be altered. When this happens, structural designers need to update their design strategies, and incentives must be put in place to retain the alignment between environmental impact and cost. Here, I assess the potential of carbon taxation to improve the structural design. I also assess the discrepancy in embodied carbon outcomes if construction costs remain constant, but the embodied carbon of materials is varied. Finally, I look at the effect of an early-stage design tool, PANDA, on embodied carbon outcomes of real projects. I find that carbon taxes need to be extremely high to have an effect, and that this effect is limited to certain types of frames. Embodied carbon in construction can become disconnected from costs if the embodied carbon of materials varies heterogeneously. Finally, novel design tools can help designers substantially improve the embodied carbon of their design. This happens despite the absence of a significant monetary incentive to that effect.

This article is part of the discussion meeting issue ‘Sustainable metals: science and systems’.

## 1. Introduction

The construction sector is responsible for perhaps as much as 30% of all CO_2_ emissions. Of these, as much as half are linked to the production of materials and the rest are operational emissions linked to heating, lighting and use of appliances. Cement alone accounts for at least 8% of all CO_${,}_{2}$_ emissions [1–3]. Steel and concrete represent the overwhelming majority of material used in construction [4].

There are therefore considerable efforts made to improve the environmental impact of these materials. New cement blends and concrete mixes promise much lowered impact [5,6], and Electric Arc Furnace (EAF) steel recycling emits much less CO_${,}_{2}$_ than BOS-produced steel [7].

Aside from the material production, policy options favouring reuse or prolonging the lives of existing structures have been suggested [8]. Finally, the third lever is improvements in design, which could reduce the embodied carbon by as much as 30% in new built structures [9]. However, the interplay between these options has not been thoroughly investigated. When a new structure is designed, it is in many cases possible to select amongst a number of structural systems, which are typically characterized by varying ratios of steel and concrete [10].

Today, the choice of the structural frame is driven by a combination of economic considerations and the availability of local expertise. As the importance of the embodied carbon grows, this choice of structural system will grow in importance [1,9]. However, as Dunant *et al*. found, savings in cost and in embodied CO_${,}_{2}$_ can be obtained together [9]. Crucially, this depends on today’s costs of frames and today’s material’s embodied carbon. Whereas the costs are likely to remain the same as the lower-carbon technologies for the production of materials are already competitive, the embodied carbon of materials is likely to change.

If steel decarbonates faster than cement, then a low-impact structure should likely be more steel-heavy until concrete improves similarly, and, conversely, if the cement producers’ decarbonation efforts bear fruit earlier, concrete construction would then be favoured. Over the next decades, this will likely change multiple times, as the transition will occur at the rate of very large plants being transformed, refurbished or closed down, causing large step changes in the evolution of the embodied carbon of materials. Therefore, it is likely not possible to provide a general guidance as to what should the optimal structural choices be over the next decades. Furthermore, in 2050, the frequent target for the transition to net zero is only 25 years away [11], which is at most two releases of the world’s main construction codes, which indicates that the current construction practices will likely stay unchanged.

Today, the amount of cement used is expected to grow by approximately 15% by 2050 [12]. Steel consumption is expected to increase much more, as much as 40% [13]. However, not all of the steel produced in the world ends up in construction. Rather, today, about 50% is used this way [14]. In 2050, as the world develops, this fraction is expected to go down: the evolution of cement consumption and steel in construction should follow similar trajectories (compare [14] for the world with [15] for the UK).

The effect of carbon taxation depends on the elasticity of the prices in real estate. The fraction of the total cost in a construction project that depends on construction proper is quite small, around 8%. Therefore using the elasticity of the property market is an adequate proxy for the elasticity of prices in construction [16]. If the elasticity is low, that is the demand is not affected significantly by price changes, then the effect of the taxation will be borne entirely by the clients, who will pay for the extra costs. However, if the elasticity is high, the building industry will try to absorb as much of the cost it can, and this will drive designs to lower-carbon solutions, in principle. Price elasticity of construction is a local variable. In some locations, construction is cheap and elsewhere it can be extremely expensive. Previous modelling found carbon taxes or carbon trading to only have a very marginal effect on output [17]. Previous research has not, to the author’s knowledge, looked at whether economic efficiency and carbon efficiency were aligned or the impact of multiple interventions when information about optimal design is imperfect.

What is the environmental cost of designers holding mistaken beliefs about the relative environmental performance of structural systems? How can new design tools and improved incentives help alleviate this? By 2050, on current trends, the world will reach its peak consumption of steel and cement in construction, and understanding these factors can have the most impact today.

## 2. Material and methods

To evaluate the impact of changes in the production system on the embodied carbon of structures, we consider likely evolutions to occur over the next three decades in cement and steel manufacturing, the increased role of steel recycling, the scale-up of the use of novel SCMs and improvements in concrete design.

*Moving steel production to EAF, DRI-EAF*: approximately 70% of steel in the world is produced through the BOS-BOF route, which uses coke as a reducing agent to produce iron from iron ore. Steel produced using this method has an embodied carbon of around 2.6 t CO_${,}_{2}$_/t of steel [18,19]. Twenty-three per cent of steel production is from recycled steel scrap in EAF [13]. Steel produced using this route can have variable embodied carbon depending on the carbon intensity of the electricity used, but is around 0.4 t CO_${,}_{2}$_/t of steel. Finally, iron ore can be reduced directly. Today, this is most often done using methane as a source for hydrogen, with an emission of 2.1 t CO_${,}_{2}$_/t of steel, but could in principle also be done using hydrogen from electrolysis. This latter option is not economically viable today.

*Novel SCMs*: today the clinker factor, that is, the fraction of cement made using Portland clinker (0.8 t CO_${,}_{2}$_/t of clinker) is limited by the availability of supplementary cementitious materials, mostly ground granulated blast furnace slag (GGBFS), a by-product of the BOS-BOF route, and Fly Ash, a residue of coal burning. This clinker factor is on average 0.73 worldwide (IEACement). However, novel developments have made it possible to manufacture SCMs in large quantities. Calcined clays have much lower embodied carbon than Portland clinker (0.3 t CO_${,}_{2}$_/t of clinker) and can be used in so-called ternary blends with embodied carbon close to 0.6 t CO_${,}_{2}$_/t of cement [5]. Therefore, despite the expected reduction in the availability of GGBFS and Fly Ash, the overall carbon intensity of cement can decrease.

*Better concrete design*: concrete mixes today use a minimum amount of cement prescribed by norms. It is however possible to make concrete mixes with high performance and lower cement, directly reducing emissions. This is done by adjusting the aggregate size distribution, reducing the mix water in cement and using admixtures to keep the flowability of concrete. This intervention can easily reduce the embodied carbon of concrete by as much as 30% [6].

Timber use as a construction material is rather marginal, and its maximum potential impact is much lower than even small tweaks in concrete mix design [6].

These interventions are all using already industrially deployed technologies, which do not require further technical developments. Applying them unequally can however considerably change the optimal mix of material within structures.

To model the structural design, we use PANDA, an automated design tool that can provide Eurocode-compliant structural frame designs using a wide range of framing options [20]. In this study, we limit the design options to steel-framed buildings, with composite concrete slabs or precast planks, or concrete-framed buildings with two-way flat slabs, ribbed or waffle slabs. The tool can optimize structures to minimize costs or to minimize the embodied carbon of the structural solutions. For the purpose of this study, the structures designed will minimize cost as much as possible. The structural systems that are considered are:

*Steel-framed precast structures*: the beams and columns are steel I-sections, but the decks are made using post-tensioned hollow-core slabs. The design considers all serviceability and ultimate limit states of the beams and columns. The decks are selected according to the manufacturer’s span tables.

*Steel-framed composite structures*: the beams and columns are steel I-section, designed if appropriate to work compositely with the concrete decks poured over thin steel profiles. The design of beams considers all appropriate checks including shear connection, and the decks are selected according to the manufacturer’s span tables.

*Two-way flat slabs*: concrete flat slabs are the preferred mode of construction due to the low cost, flexibility of design and familiarity. Further, their geometry makes service integration easy, helping specialization and further reducing costs. In the model, the slabs are designed according to the Eurocode (BS EN 1992-1-1, Eurocode 2), with the reinforcement scheme designed following a simple moment strip approach. Jayasinghe *et al.* looked at the effect of tweaking the design of flat slabs with fixed carbon factors and found some potential for savings [21].

*Waffle slabs*: deep concrete slabs, hollowed in their underside to only keep the concrete ribs required for the steel offer excellent structural efficiency at the cost of reduced flexibility. There exists specialized formwork solutions in markets where such structural forms are favoured that make these systems cost-competitive. At the expected cost per square metre of formwork, they remain competitive even where they are uncommon. The key drawback is that they only allow for specific bay shapes, and this may not be acceptable if the layout of the building is complex.

*Ribbed slabs* are a compromise between the flat slab and the waffle slab, where the concrete ribs are in a single direction. Like the waffle slab, they only work on a restricted set of dimensions. For both beams and waffle slabs we consider the beams as integral to the design of the slab.

To study the drivers of change, we use two separate interventions. The first looks at the introduction of a carbon tax, and its influence on the choice of structural form. To do this, we use PANDA [9,22] to consider such a tax in the design automatically. We then select cost-optimal solutions and compare them across levels of taxation. Two 36 m × 24 m buildings with a basement were used as model structures; one is six storied and the other 16 storied. The range of spans in both directions goes from 3.5 to 12 m. Only steel and concrete structural solutions are considered, as these two materials represent almost all the available materials for construction. The carbon factors used for the materials are representative of the world average carbon factors (table 1). The loads considered were 2.5 kN/m${,}^{2}$ superimposed and dead loads except for the roof where the imposed load is 0.75 kN/m${,}^{2}$. Total, dead and imposed load deflection limits were set at 1/250, 1/200 and 1/360, respectively. A simple vibration check was applied on beams, which were required to have a natural frequency higher than 4 Hz. For the design of foundations, the floor was assumed to be made of clay with a 75 kN/m${,}^{2}$ undrained shear strength.

**Table 1.** Carbon intensity factors used for the analysis of the structures generated with PANDA. These values reflect world averages. The minimum values were obtained by applying efficiency factors to steel and concrete as discussed in the text, leaving the other materials used in steel and concrete construction untouched.

material	CO_2_ intensity (cradle to gate) kg CO_2_/kg	minimum CO_2_ intensity (cradle to gate) kg CO_2_/kg
concrete C30/37	0.1408	0.041
concrete C32/40	0.1514	0.042
concrete C35/45	0.1621	0.043
steel (rebars)	1.55	0.1
steel (deckings)	2.76	0.1
steel (beams)	1.99	0.1
plywood (formwork)	0.681	0.681
paint (primer)	3.530	3.530
paint (topcoat)	3.700	3.700
paint (intumescent)	2.529	2.529

The second driver of change we considered is the availability of improved design methodologies and tools. The embodied carbon of structures designed with and without PANDA has been recorded over 3 years at a London structural design firm. The database contains 400 structures, with the frame types, materials quantities, purpose and massing information. We could then quantify the effect of using better early-stage design selection methods on the final carbon of realized projects.

Finally, to evaluate the cost of changing today’s alignment between construction costs and carbon factors, we consider the same massing as above, but using different carbon factors. At the technological limit, the steel could be produced entirely in EAFs, using decarbonated electricity. The amount of lime required in the process, as well as the carbon electrode consumption, puts the minimum emissions of steel at 100 kg CO_${,}_{2}$_/kg. Concrete can be made using higher substitution cement. At scale, this would be done using calcined clays, with a real-world abatement of 30%. The amount of cement used in concrete mixes can also be reduced, leading to a similar reduction. Finally, efficiencies in the materials burnt for the calcination, recycling, etc., can lead to an overall abatement of 70%. We test how the embodied carbon of the model structures differ when the cost of each frame remains constant.

## 3. Results

I present first the carbon impact of frames under extreme abatement scenarios using today’s technologies. The effect of carbon taxation is then evaluated using the same model structures. Finally, the impact of the introduction of a novel design tool is presented.

**Figure 1.**
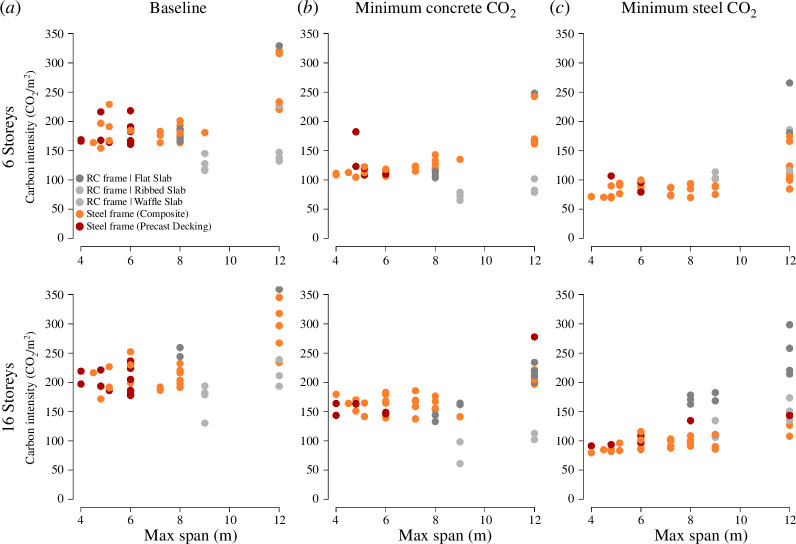
Changes in frame selection under a number of scenarios. The top row are six-storey structures with a reinforced concrete basement; the bottom row are 16-storey structures with a reinforced concrete basement. (*a*) World average carbon factors. (*b*) Minimum concrete CO_${,}_{2}$_ factors and world average steel. (*c*) Minimum steel and average concrete figures. Multiple designs are produced for each span, corresponding to the column spacing in the transverse direction.

The most economic frame varies as a function of the span, more than as a function of the structural height (figure 1*a*). In general, the embodied carbon grows with the span, except when waffle or ribbed slabs are designed. These can be efficient only beyond certain spans, and at certain bay aspect ratios. The frames with the lowest carbon footprint depend on the embodied carbon of steel versus concrete. This is visible when comparing the middle and rightmost columns of figure 1. Improving the carbon content of concrete can have the most impact in frames with mid-sized spans (7–9 m) whereas improving the footprint of steel can enable long-spanned frames with relatively low embodied carbon. In this set of experiments, the aspect ratio of bays can be large, therefore the highest embodied carbon recorded for each span is likely more representative of the effect of span. Nonetheless, this illustrates that the overall massing and layout of a structure can create efficiencies or inefficiencies. In general, designing to the ‘wrong’ material abatement can increase the embodied CO_${,}_{2}$_/m${,}^{2}$ of frames by 50–75 kg CO_2eq_. This is one band in the IStructE CORS rating scheme. Can a change in financial incentives help retain alignment between cost and carbon?

**Figure 2.**
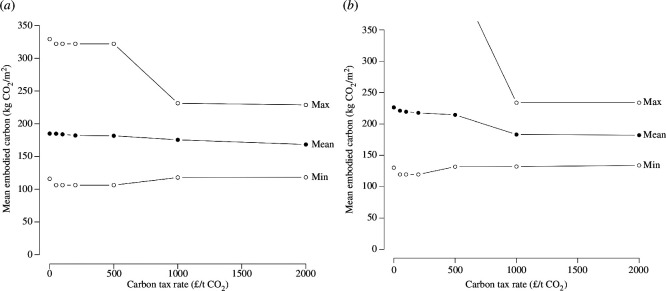
Changes in minimum, maximum and mean carbon intensity of generated structures under a range of carbon tax rates assuming world average carbon values for steel and concrete. (*a*) Six-storey structure with a basement. (*b*) Sixteen-storey structure with a basement. The effect of the tax is minimal, because the embodied carbon and cost are well aligned.

The application of a carbon tax raises the price of all structures, with the least efficient ones being more affected. This causes the least efficient structures to become too expensive or change their design, in principle (figure 2). However, looking only at the cheapest option for each layout, even very high carbon taxes cannot change the optimal choice of material, only minor aspects of detailing, sometimes even adversely affecting the carbon rate. Small changes in the carbon rate are due to slightly different optimal reinforcing layouts under different tax regimes. This is because the choice of the most cost-effective structure typically coincides with the choice of the most carbon-effective one. Indeed, the minimum carbon intensity does not vary with the change in tax rate. The essential difference is in the choice of the foundations: where piles or rafts are required because shallow foundations like pads are not possible, the carbon tax may make the choice of a raft uneconomical, and rafts are commonly more carbon intensive than piles. This mostly affects the most carbon-intensive structures.

This effect of taxation on the carbon intensity of structures can change if the relative carbon intensity of materials is modified: although it is in principle possible in concrete construction to trade the depth of the slabs with steel reinforcement, steel and carbon frames have different carbon/cost trade-offs. Because of that, carbon taxation will impact differently the steel and cement sectors.

**Figure 3.**
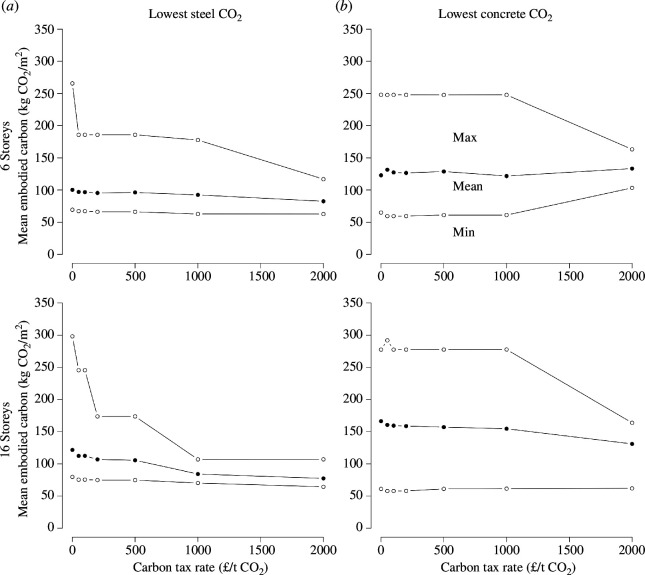
Effect of a carbon tax for two building typologies, assuming decarbonation of the steel or cement sectors only to today’s known technological frontier. (*a*) This scenario assumes that steel is produced following the best available technology, and has the lowest CO_2_ impact possible today. (*b*) This scenario assumes that concrete is produced following the best available technology, and has the lowest CO_2_ impact possible today.

If the steel sector were to decarbonize faster than the cement sector, the relative role of cement in the embodied carbon of structures would increase. This would lead to an increased potential impact of carbon taxation, with even small taxes reducing the embodied carbon of the least carbon-efficient structures (figure 3*a*). On the contrary, cement decarbonation does not change the impact of carbon taxation, which remains ineffective at improving outcomes.

Even though frame design is relatively insensitive to carbon taxation, when the embodied carbon of concrete and steel are varied, the optimal frame design in terms of carbon changes. Structural designers, although they design economically efficient frames, are also motivated to reduce the embodied carbon of their design. However, a rapid change in the carbon factors of materials complicates the designer’s work. For example, if concrete were commonly produced to the state of the art, then concrete frames would typically be less carbon intensive than steel frames. Furthermore, the effect of increasing the recycling fraction of steel will likely first impact reinforced concrete frames as reinforcing steel has the least restriction to its quality. However, structural steel members are also commonly made with relatively low-grade steel, and an abundant supply of recycled steel will first lower the CO_2_ of the construction sector. These effects make it more difficult for designers to keep current on the embodied carbon consequences of their choice of structural frame. To help with this choice, a structural design tool, PANDA, is used.

PANDA has been deployed at a London mid-sized structural engineering practice since 2019, Price & Meyers. This practice had been tracking their embodied carbon since before the deployment (figure 5). Since all structures designed at this firm face the same general incentives, figure 5 indicates that improved design tools can make a significant difference to the quality of design, and that voluntary change can be effective. All designs have improved, indicating a drive to lower-carbon design, but the designs using PANDA have even lower embodied carbon. How have the engineers reduced the embodied carbon of their designs? Two possible mechanisms could explain this result: the designs guided by PANDA are better, or the designer chose lower-carbon materials for their designs at a higher frequency (possibly under the guidance of PANDA).

**Figure 4.**
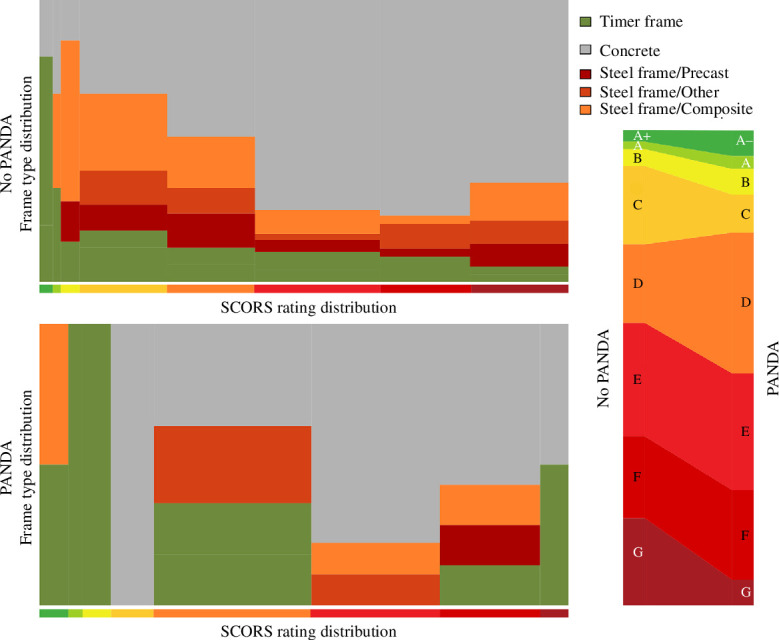
Distribution of the frame types and CO_${,}_{2}$_ rating in the dataset, restricted to structures that can be designed with PANDA. The width of the bar indicates the proportion of structures in each bin. A key feature of this dataset is that all frame types can be seen in all bins, in relatively similar proportion. The bins correspond to the IStructE’s SCORS scheme [23].

**Figure 5.**
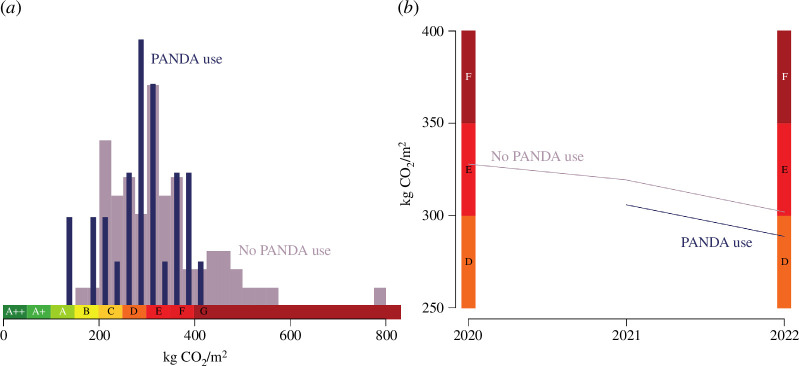
Relative distribution of ratings depending on whether PANDA was used (*a*). Trend in the embodied carbon in the dataset (*b*): average value weighted by floor area. PANDA use correlates with lower embodied CO_${,}_{2}$_, and this effect is predominantly linked to avoiding very high-intensity projects, as well as improving the designs. The bins correspond to the IStructE’s SCORS scheme [23].

The structures designed with the tool have a similar distribution of structural frame material choices (figure 4). However, they are systematically lower carbon. Although lower-carbon structures are more commonly timber-framed structures and higher embodied carbon structures are more commonly concrete-framed ones, there are structures of all CORS rating for every frame type. This indicates the frame selection is really dependent on the specifics of each project. This suggests that the quality of the design, with respect to emissions was improved by the tool. The way in which the designs are improved seems to be mostly by shifting the outcomes (figure 5*a*). Crucially, the tool seems to avoid the design of very high embodied carbon frames.

Overall, there is a learning curve visible in the dataset: the embodied carbon of all structures at Price & Meyers is reducing over time as a result of the voluntary policy put in place.

## 4. Discussion

The cost incentives in construction depend on the elasticities. A number of studies have looked at the historical trends in elasticities of prices in construction in a number of markets [24–26]. The elasticity varies quite a bit from very elastic to completely inelastic depending on the geographic location, and depends on geographic, institutional and economic factors [26]. Therefore, the effect of increasing the price of construction, e.g. through taxation, may decrease construction overall when the elasticity is high. Jochem *et al.* looked specifically at the price elasticity of timber, a commonly proposed low-carbon alternative construction material [27]. They find that estimation is not possible, consistent with the findings in this work: the choice of material is not affected by adding a carbon price, and therefore no material should see its use vary as prices change. Pryce finds that holding land vacant when there is uncertainty in how it will be used when developed increases the inelasticity of prices [28]. This indicates that if low-carbon construction is favoured, uncertainty in how it should be achieved should increase construction prices. In general, the literature suggests that increased carbon taxation in construction is unlikely to alter the design of structures. Rather, it may decrease the output of construction in markets where the elasticity is high enough, and will decrease social welfare in inelastic markets. This is consistent with the results presented here, which further suggest that the reason for the non-transmission of incentives is the structural engineers already produce cost *and* carbon-optimized designs.

Policies aiming to favour specific frame types will likely result in disappointing impact. This is because although certain frame types are more likely to lead to lower-carbon buildings, any frame type can be used to design a low-carbon building, and crucially, any frame type can result in a high embodied carbon building. It is the efficient use of structural materials that drives the reduction in CO_${,}_{2}$_. Importantly, we find that carbon taxation can have an effect only when steel decarbonates faster than cement. This is because steel is more expensive than cement, and taxing carbon can shift more carbon-intensive concrete frames to steel frames. This effect is however limited as it only concerns the most carbon-intensive structures. Overall, both interventions, carbon taxation and favouring certain frame types, are not suitable to reduce carbon in construction without either little effect or loss of welfare. This is because the challenge of low-carbon design is to match the correct frame design to the specifics of each project.

Matching the project with the ‘best’ frame using outdated carbon factors can lead to as much as 50 kg of CO_2eq_ more per m${,}^{2}$ to be embodied in the frame. As material production is rapidly changing, keeping guidance current is critical and the use of correct carbon factors for each project is necessary.

Tracking the effect of introducing PANDA in the engineer’s practice over time shows the potential for better design in embodied carbon outcomes. About half of the potential improvement (30%) suggested by Dunant *et al.* [9] could be realized in 2 years. A strategy to lower carbon through better design is not instantly effective because new practices have to be learnt over time. The learning effect is visible with respect to the tool, the effect of which is increasing over time: the ratio of PANDA structures’ embodied carbon over the non-PANDA structures is decreasing. This shows that even with an available tool that guides optimal design choices, the design habits in construction are slow moving, and this can have important consequences when the ratio of embodied carbon between materials changes quickly. The main effect of having a tool that enables better early-stage design is that the worse outcomes in terms of embodied carbon seem to be avoided. The second visible effect is that designs seem to be biased towards the D band of the IStructE SCORS system [23], whereas without PANDA no clear separation between D and E band is visible. This may be explained by incentives to meet thresholds.

Decarbonating steel and concrete face different industrial challenges: although they both are capital-intensive industries, steel is more so than concrete, meaning decarbonation can happen very quickly if a decision is made to replace blast furnaces with EAFs, but similarly, this transition can be delayed depending on the political environment. Concrete decarbonation can be done much more progressively, owing to the more decentralized nature of production and the availability of a larger range of decarbonation options.

## 5. Conclusion

This article identified the importance of considering construction as a ‘composite’ problem. In construction, there are no sustainable materials, rather there are sustainable practices. The design of structures optimized for cost is not sensitive to carbon taxation, therefore this cannot be used as a tool to incentivize low-carbon construction. Which structures are carbon efficient is quite closely aligned to which structures are cost-efficient today. This may change as the production of steel or concrete is decarbonated without the other material being similarly improved. In this case, informing engineers is the key mechanism to improve outcomes. The importance of information was validated by looking at real-world deployment of a software tool guiding the initial choice of frame in structural design: the adoption of the tool led to a reduction of 15% of the embodied carbon within 2 years when it was deployed.

## Data Availability

The data used for the analysis can be found in the supplementary information [29].
